# Real-world management of achalasia and esophagogastric junction outlet obstruction in Italy: results from a national survey

**DOI:** 10.1007/s13304-025-02406-8

**Published:** 2025-09-24

**Authors:** Elettra Ugliono, Salvatore Buscemi, Danilo Consalvo, Angelo Iossa, Nicola Tamburini, Graziano Pernazza, Fabrizio Rebecchi

**Affiliations:** 1 General Surgery and Center for Minimally Invasive Surgery, Department of Surgical Sciences, University of Turin, Corso A.M. Dogliotti 14, 10126 Turin, Italy; 2 Azienda Di Rilievo Nazionale Ad Alta Specializzazione Ospedali Civico Di Cristina Benfratelli, Palermo, PA Italy; 3 Department of Gastroenterology and Digestive Endoscopy, AORN “Antonio Cardarelli”, Naples, Italy; 4 Department of Medico Surgical Sciences and Biotechnologies Sapienza Polo Pontino, ICOT Hospital Latina, Latina, Italy; 5 Department of Thoracic Surgery, Azienda Ospedaliero Universitaria Di Ferrara, Ferrara, Italy; 6 General and Robotic Surgery Department, AO San Giovanni Addolorata, Rome, Italy

**Keywords:** Achalasia, Heller myotomy, EGJOO, Esophago-Gastric Junction Outflow Obstruction, POEM

## Abstract

There is significant variability in clinical guidelines for achalasia, and precise indications for Esophagogastric Junction Outflow Obstruction (EGJOO) are lacking. The recommendations provided in the published literature could be difficult to translate into the clinical practice due to the discrepancy in the available resources. This survey aims to provide insight into the different diagnostic and therapeutic approaches adopted nationwide. An electronic 31-item questionnaire was sent among the members of the Italian Society for Endoscopic Surgery of Endoscopic Surgery and New Technologies (SICE). A single response from each participating center was required. A total of 46 answers were obtained. The first approach to achalasia was Heller myotomy plus Dor fundoplication (H–D) in most cases, but there was an increased use of Per-Oral Endoscopic Myotomy (POEM) for subtype III achalasia. Botulin toxin injection (BTX) and PD were reserved for frail, older patients. Surgery was the primary approach for end-stage achalasia, mainly H–D (50.0%), esophagectomy (22.7%), and PD (20.5%). A conclusive diagnosis of EGJOO was managed through PD (32.6%), clinical observation (21.7%), H–D (17.4%), Proton Pump Inhibitors (PPIs) (13.0%), BTX (13.0%) and POEM (2.2%) while an inconclusive EGJOO diagnosis through clinical observation (39.1%), PD (23.9%), H–D (21.7%), PPIs (8.7%) and POEM (6.5%). The suggested timing was 3 months (72.7%) for clinical and 6 months (63.6%) for instrumental follow-up. In case of persistence of symptoms, the preferred treatments were H–D (50.0%) and PD (28.3%). This study provides a real-world snapshot of the management of achalasia and EGJOO in the Italian landscape, showing a wide variability in the clinical practice among the involved centers. A multidisciplinary approach is advisable, and clinical guidelines are warranted to provide shared decisions for the management of these disorders.

## Introduction

Achalasia spectrum disorders are a subset of esophageal dysmotilities that share an impaired Lower Esophageal Sphincter (LES) relaxation as a common element. The Chicago Classification version 4.0 (CCv4.0) recognizes, according to High-Resolution Esophageal Manometry (HREM) peristaltic pattern, four possible phenotypes: absence of peristalsis (type I achalasia), pan-esophageal pressurizations (type II achalasia), premature contractions (type III achalasia) and intact peristalsis (Esophago-Gastric Junction Outlet Obstruction, EGJOO) [1].

Several guidelines have been developed to provide recommendations for the management of achalasia [2–4]. However, significant variability exists regarding the content and strength of their statements on the diagnosis and treatments of achalasia. Huang et al. performed a comprehensive study evaluating systematically the methodological quality of guidelines for the diagnosis and treatment of achalasia, using the Appraisal of Guidelines for Research and Evaluation II (AGREE II) instrument. Among the seven guidelines published between 2018 and 2021, the overall quality assessment was modest, ranging from 48.3% to 68.1%. The lowest values were observed in the “applicability” domain, with a median score of 41.8% (35.4–57.3%), and “stakeholder involvement” domain (median score 45.4%, ranging from 20.8% to 73.6%), with only two guidelines taking into account patients’ perspectives and expectations. According to the authors, the primary reasons for this lack of consistency included methodological flaws in literature searches, heterogeneity in the strength of recommendations, and variation in local resources, infrastructure and current practices considered by the guideline development groups [5]. These results highlight the challenges involved in implementing guideline recommendations in the clinical practice. On the other hand, there are no established guidelines for the management of EGJOO patients, leaving the involved clinicians with limited direction for clinical decision-making. Furthermore, the continuous introduction of new and increasingly sophisticated diagnostic tools, along with the emergence of novel advanced therapeutic approaches, have led to significant variations in clinical practice among different centers, depending on the availability of equipment, resources, and infrastructures.

The aim of this study is to perform a real-life snapshot of the management of achalasia and EGJOO in the national landscape, in order to inform on the different diagnostic and therapeutic approaches adopted nationwide, highlight unmet clinical needs, and stimulate the discussion among the clinicians involved in the management of these disorders.

## Materials and methods

For this observational cross-sectional survey study, an electronic 31-item questionnaire was developed among the “Benign Upper Gastrointestinal Research Group” of the “Italian Society for Endoscopic Surgery of Endoscopic Surgery and New Technologies” (SICE) via Google Forms. The survey was conducted in accordance with international guidelines for reporting results of internet E-Surveys (CHERRIES) [6].

The survey is the first step of a multicenter project aiming to establish a National registry for achalasia spectrum disorders. Each participating center, which expressed willingness to proceed with phase 2 of the project, was requested to submit a single response to the survey.

A first pilot version was tested among the Research Group members to assess the technical functionality of the electronic questionnaire. The final version was uploaded to the official SICE website, distributed via a mailing list to all social members, and advertised on SICE official social media. The survey was open to all involved clinicians, independent of social membership, above voluntary participation, from June 1 st to September 30th, 2024.

The questionnaire consisted of 31 items, divided into three sections: Q1-Q5, general information about the facilities available at the responder’s working center; Q6-Q16 questions about the diagnostic and therapeutic management of achalasia subtypes; Q17-Q31 questions about the clinical management of EGJOO. The answer was mandatory to multiple choice and Likert-scale questions, optional for open questions.

### Statistical analysis

After the closing date of the questionnaire, the data were downloaded and analyzed via STATA software version 18. Descriptive statistics were used to report the findings of the survey. The results were summarized as numbers (n) and percentages (%). Results were reported as mean and standard deviation for questions that could be quantified.

## Results

A total of 46 answers were obtained. The response rate was not calculated since the exact extent of the dissemination of the questionnaire was not evaluable.

### Section I – Questions Q1-Q5

**Fig. 1:**
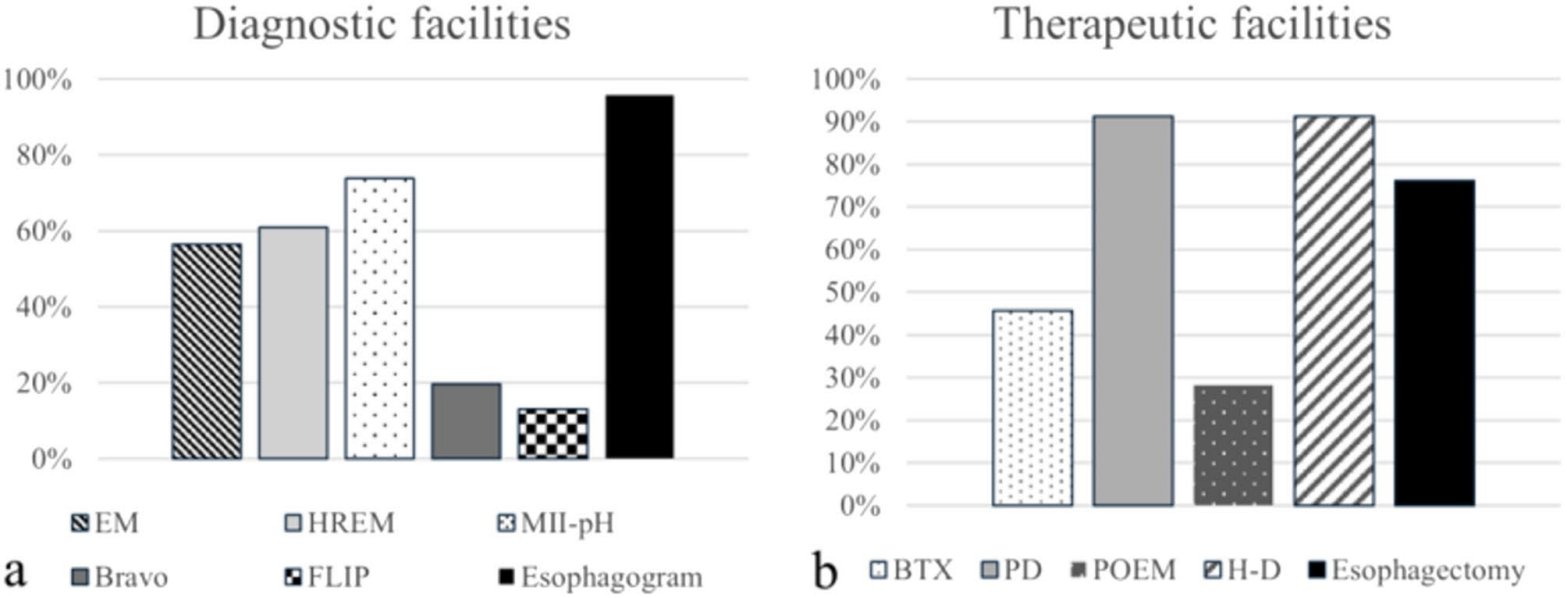
Diagnostic (**a**) and therapeutic (**b**) facilities available at each participant center. *EM* conventional Esophageal Manometry, *HREM* High-Resolution Esophageal Manometry, *MII-pH* 24 h intra-luminal pH-impedance monitoring, *Endo-FLIP* Endoluminal Functional Lumen Imaging Probe, *BTX* endoscopic Botulin Toxin Injection, *PD* pneumatic dilatation, *POEM* Per-Oral Endoscopic Myotomy, *H–D* Heller myotomy plus Dor fundoplication

Questions Q1-Q5 assessed the available facilities and volume of patients of each participant center. The primary featured diagnostic facilities were RX esophagogram, which was quite ubiquitous (n = 44, 95.7%), followed by 24-h intra-luminal pH-impedance monitoring (MII-pH), (n = 34, 73.9%). High-Resolution Esophageal Manometry (HREM) (n = 28, 60.9%) and conventional Esophageal Manometry (EM) (n = 26, 56.5%) were present in more than half of the participant centers, while 48-h Bravo capsule pH monitoring system (n = 9, 19.6%) and endoluminal Functional Lumen Imaging Probe (FLIP) (n = 6, 13.0%) were less commonly present (Fig. 1a).

Pneumatic Dilatation (PD) (n = 42, 91.3%) and Heller myotomy plus Dor fundoplication (H–D) (n = 42, 91.3%) were frequently performed, while esophagectomy (n = 35, 76.1%) was limited to referral centers. A minor role was left to endoscopic Botulin Toxin injection (BTX) (n = 21, 45.7%) and Per-Oral Endoscopic Myotomy (POEM), available in 28.3% of centers (n = 13) (Fig. 1b).

When asked about patient volume, the annual number of patients diagnosed with achalasia was “less than five” in 17 (37.0%), “5–10” in 14 (30.4%), “10–20” in 5 (10.9%) and “more than 20” in 10 (21.7%) centers. The number of patients diagnosed with EGJOO each year was lower, specifically “less than five” in 24 (52.2%), “5–10” in 14 (30.4%), “10–20” in 4 (8.7%), and “more than 20” in 4 (8.7%) centers.

### Section II – Questions Q6-Q16

Questions Q6-Q16 assessed the diagnostic and therapeutic management of achalasia at each participant center.

**Fig. 2:**
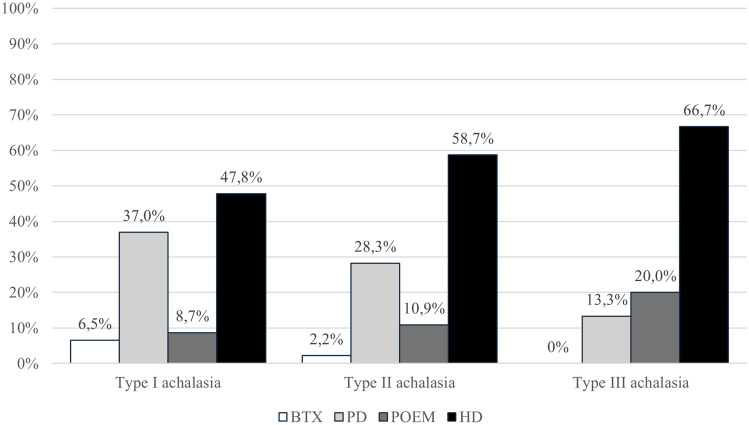
First therapeutic approach to achalasia according to subtype. *BTX* endoscopic Botulin Toxin Injection, *PD* pneumatic dilatation, *POEM* Per-Oral Endoscopic Myotomy, *H–D* Heller myotomy plus Dor fundoplication

The results of the survey indicated clinical differences in the initial therapeutic approach to achalasia depending on its subtype. Although the first approach of choice to achalasia remains H–D in most cases, there was evidence of increased use of POEM over PD for subtype III achalasia compared to subtypes I-II, where PD was the second main therapeutic option. Figure 2 summarizes the results.

When asked about the optional technical modifications that can be performed during the H–D procedure, 11 respondents (23.9%) declared to perform a myotomy elongation in type III achalasia, eventually guided by intraoperative manometry, and four (8.7%) chose to perform a Toupet over a Dor fundoplication in selected cases, such as the presence of a concomitant hiatal hernia.

In the case of a diagnosis inconclusive for achalasia, defined as the absence of esophageal peristalsis without impaired LES relaxation, the first approach consisted of pharmacological therapy (n = 23, 50%) and clinical observation (n = 16, 34.78%), while a minority of centers indicated to perform endoscopic or surgical procedures (BTX n = 2, 4.4%; PD n = 4, 8.70%; H–D n = 1, 2.2%).

Among the respondents who reported using BTX (n = 22, 47.8%) and PD (n = 43, 93.48%) as a treatment modality for achalasia, the main indications were frail patients with significant anesthesiologic comorbidities (BTX 81.8%, PD 79.1%), elderly patients (BTX 72.7%, PD 48.8%), bridge to surgery (BTX 36.4%, PD 46.5%) and patients with previous esophagogastric surgery (BTX 18.2%, PD 53.5%).

**Fig. 3:**
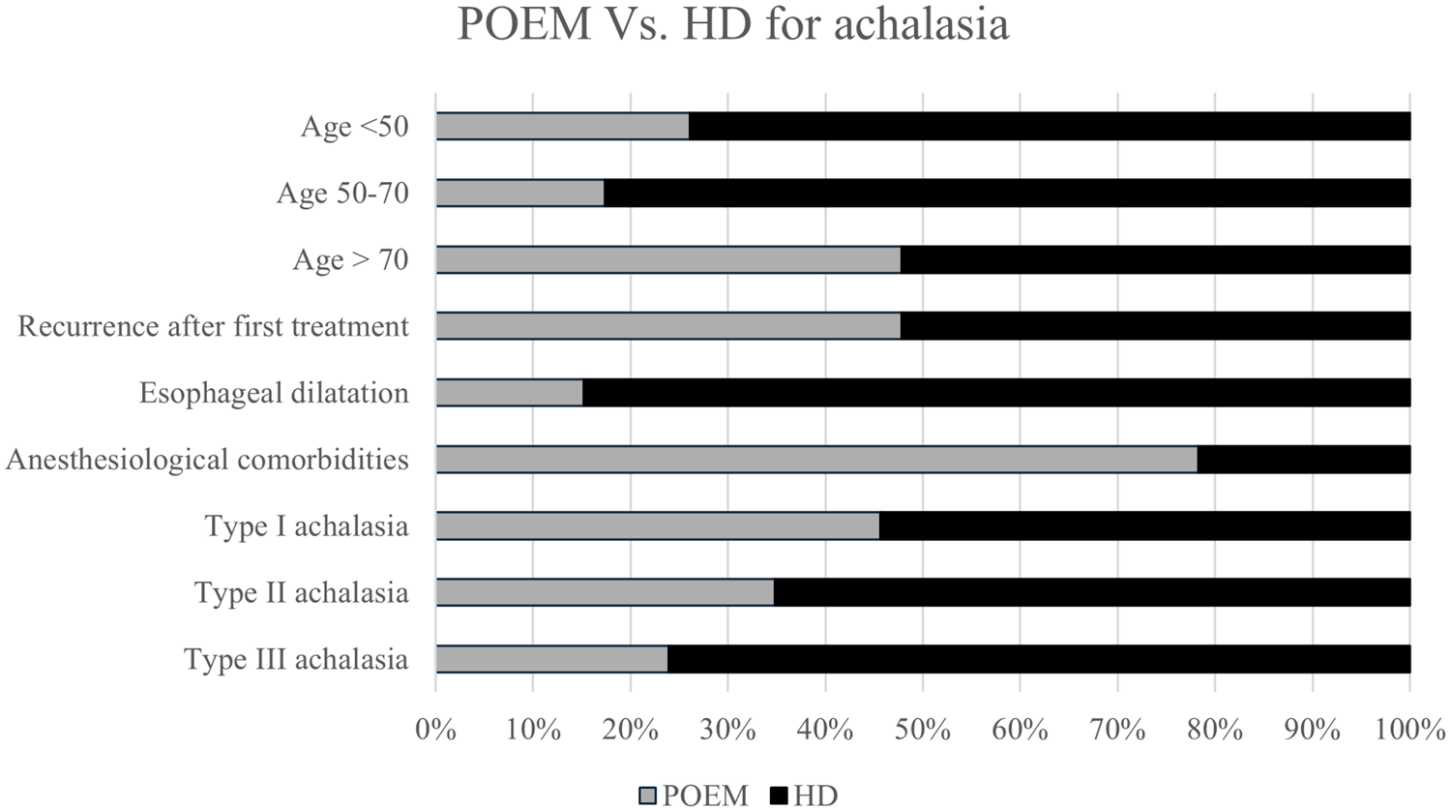
Comparison between POEM and Heller-Dor indications for achalasia. *POEM* Per-Oral Endoscopic Myotomy, *H–D* Heller myotomy plus Dor fundoplication

When directly compared, H–D was generally indicated more frequently than POEM as the choice procedure for achalasia treatment, except for patients with important anesthesiologic comorbidities, where POEM was preferred. POEM is considered to have a similar role to H–D for elderly patients and in case of recurrences after previous treatments. Furthermore, POEM was more frequently indicated in type I and II compared to type III achalasia. Figure 3 shows the comparison of indications for H–D Vs. POEM for achalasia.

Surgery was considered the primary first approach for end-stage achalasia with megaesophagus, with the main suggested options being H–D (n = 22, 50.0%), esophagectomy (n = 10, 22.7%) and PD (n = 9, 20.5%). POEM for end-stage achalasia was considered only by 6.8% (n = 3) of clinicians. Heller-Dor was indicated as the first standard surgical procedure for end-stage achalasia for 47.7% of respondents (n = 21), while other indications to choose for H–D in end-stage achalasia were recurrence of symptoms (n = 20, 45.5%), absence of clinical benefits (n = 18, 40.9%), and recurrence of complications after previous treatments (n = 11, 25.6%). When asked about potential indications for a resective surgery of the distal esophagus for achalasia, the most common answers were: end-stage achalasia with mega-esophagus, especially in cases of failure of previous endoscopic and H–D procedures (n = 15, 32.6%), concomitant neoplastic comorbidities (n = 3, 6.5%) and complications of prior surgery (n = 1, 2.2%).

### Section III – Questions Q17-Q31

Questions Q17-Q31 assessed the diagnostic and therapeutic management of EGJOO at each participant center.

According to the results of the survey, a conclusive diagnosis of EGJOO, which fulfilled all clinical, manometric, and instrumental criteria, was uncommon, reported to be less than 25% of patients for the majority of the respondents (77.8% using Chicago Classification version 3.0 and 89.1% using CCv4.0 criteria).

The main radiological findings that were considered suggestive for the diagnosis of EGJOO included the presence of esophageal retention of contrast medium above the esophagogastric junction (n = 28, 60.9%), narrowing of the medium passage at the esophagogastric junction (n = 27, 58.7%) and esophageal dilatation (n = 23, 50.0%). Additional possible indicators included the evidence of dysmotility, such as corkscrew esophagus (n = 17, 37.0%), the measurement of the height of the medium column (n = 9, 19.6%), and the presence of epiphrenic ampulla dilatation (n = 4, 8.7%).

The FLIP criteria considered suggestive for the diagnosis of EGJOO were EGJ-Distensibility Index < 2 mm2/mmHg for 5 (10.9%) and EGJ-Distensibility Index < 3 mm2/mmHg for 4 (8.7%). However, the majority of the clinicians (n = 37, 80.4%) did not answer the question since the device was not available in their center.

According to the experience of the participants of the survey, provocative manometric maneuvers, such as multiple rapid swallow test and rapid drinking test, were performed “always” (n = 8, 17.4%), “often” (n = 14, 30.4%), “occasionally” (n = 12, 26.1%), “rarely” (n = 8, 17.4%), “never” (n = 3, 6.5%), “unknown” (n = 1, 2.2%).

**Fig. 4:**
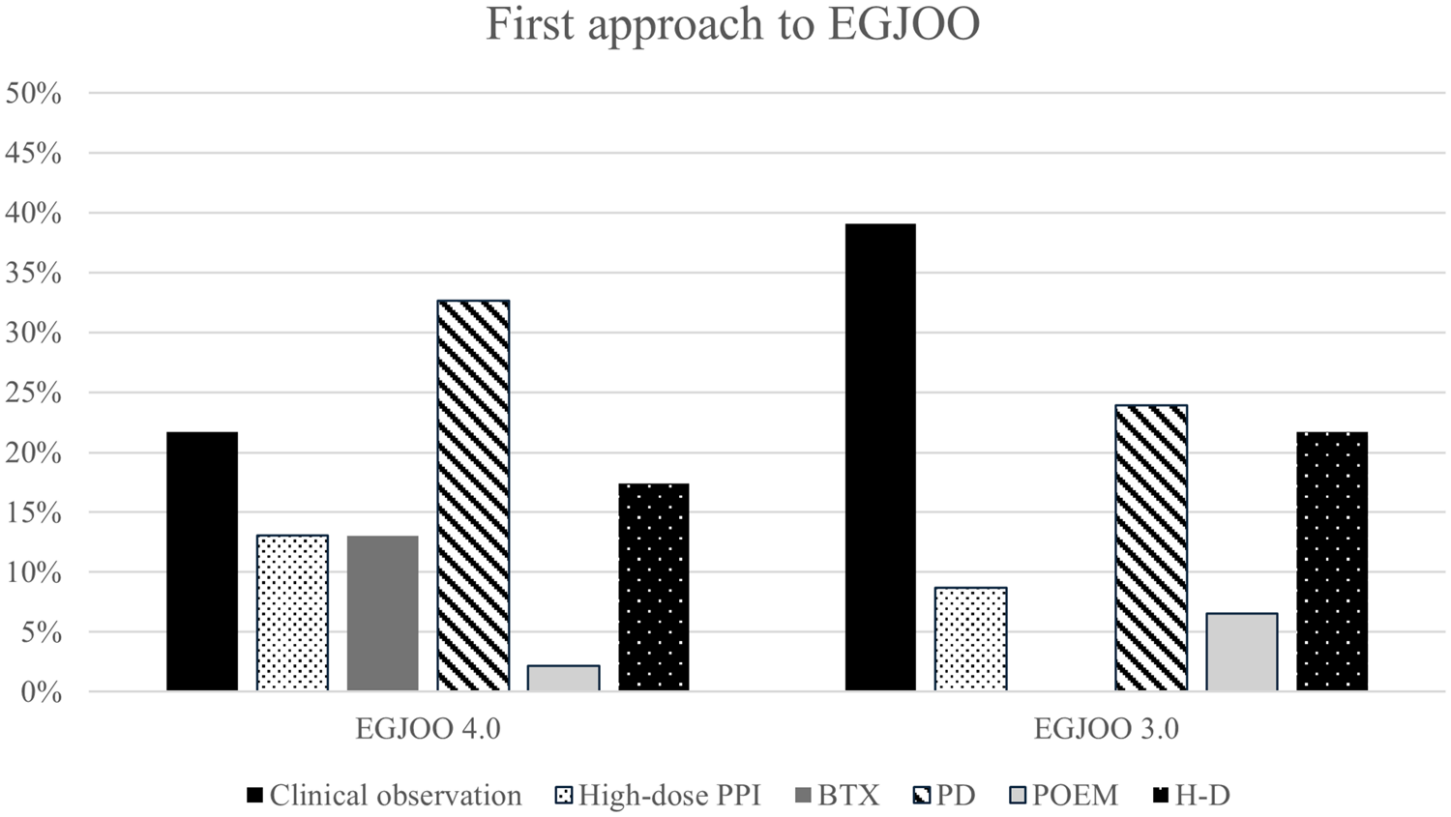
First approach to EGJOO according to manometric criteria. *EGJOO* Esophagogastric Junction Outflow Obstruction, *PPI* Proton Pump Inhibitors, *BTX* endoscopic Botulin Toxin Injection, *PD* pneumatic dilatation, *POEM* Per-Oral Endoscopic Myotomy, *H–D* Heller myotomy plus Dor fundoplication

In case of a conclusive diagnosis of EGJOO according to the CCv4.0 criteria, the first approach consisted of PD for 15 (32.6%), clinical observation for 10 (21.7%), H–D procedure for 8 (17.4%), pharmacological therapy with Proton Pump Inhibitors (PPIs) for 6 (13.0%), BTX for 6 (13.0%) and POEM for 1 (2.2%). In the case of a diagnosis of EGJOO according to Chicago Classification version 3.0 criteria, associated with clinical symptoms and abnormal radiological findings, who did not fulfill the manometric CCv4.0 criteria, the first approach was clinical observation for 18 (39.1%), PD for 11 (23.9%), H–D for 10 (21.7%), medical treatment with PPIs for 4 (8.7%) and POEM for 3 (6.5%). The results are represented in Fig. 4.

Among the 22 (47.8%) clinicians who declared to perform a conservative approach (clinical observation or PPI treatment) as the first attempt to inconclusive EGJOO, the timing for clinical reassessment was suggested to be at 3 months by 72.7% (n = 16), at 6 months by 22.7% (n = 5) and at 9 months by 4.5% (n = 1) of the respondents. The most convenient timing for the instrumental follow-up was considered to be at 6 months for 14 (63.6%), at 9 months by 1 (4.55%), at 12 months by 2 (9.09%), and variable depending on the clinical course by 5 (22.73%) participants. The instrumental reevaluation should include HREM for 67.4% (n = 31), upper endoscopy for 37.0% (n = 17), and RX esophagogram for 58.7% (n = 27) of the respondents. In case of persistence of the clinical scenario after adequate clinical and instrumental follow-up, the preferred treatment options were H–D (n = 23, 50.0%) and PD (n = 13, 28.3%), followed by BTX (n = 4, 8.7%), POEM (n = 4, 8.7%) and PPIs therapy continuation (n = 2, 4.3%).

When asked about the most appropriate indications for H–D in EGJOO, a total of 21 (45.6%) did not answer, while among the other 25 (54.3%) there was extreme heterogeneity of responses. The majority (n = 11, 44.0%) stated that the surgical indication is guided by the clinical scenario, and H–D is indicated in the presence of severe disabling symptoms. Failure of previous treatments was similarly judged to be a suitable indication for H–D in EGJOO by 6 (24.0%) respondents. On the other hand, 3 participants (12.0%) stated they would never perform H–D for EGJOO, and 1 reported to perform H–D almost always (4%). Other reported indications were young patients (n = 2, 8.0%) unavailability of endoscopic facilities (n = 1, 4.0%) and choice of the patient (n = 1, 4.0%).

**Fig. 5:**
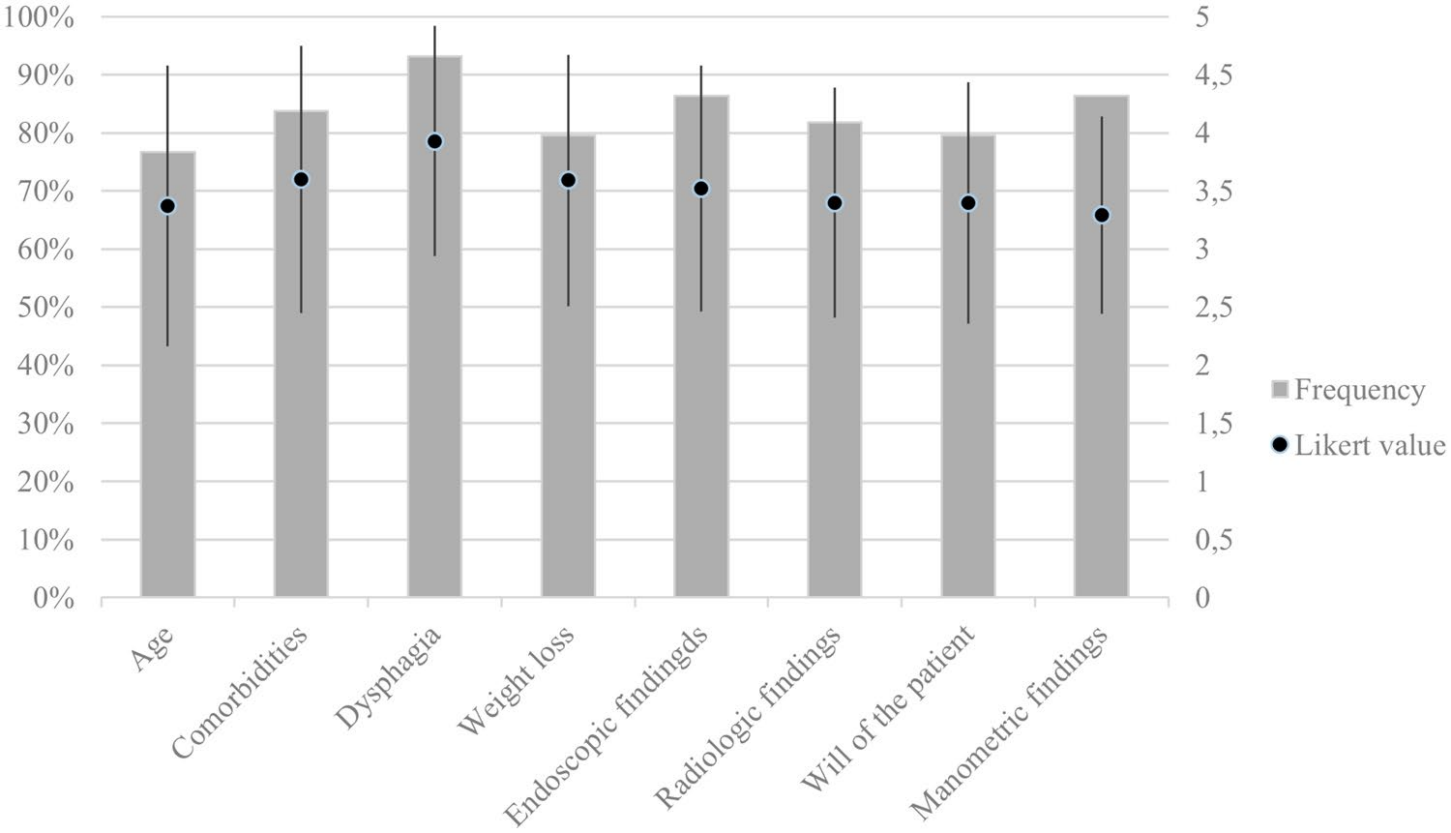
Factors involved in the choice of interventional procedures for EGJOO

Regarding the factors taken into account to guide the choice to perform interventional (endoscopic or surgical) procedures for EGJOO, the most frequently indicated are: the severity of the dysphagia (n = 41, 93.2%), the severity of the endoscopic findings (n = 38, 86.4%), and the absence of peristaltic reserve at provocative maneuvers during HREM (n = 38, 86.4%), while the most impactful factors for clinical decision, associated with the highest scores, were severity of dysphagia (3.93 ± 0.99), the presence of comorbidities (3.60 ± 1.15), and the degree of weight loss (3.59 ± 1.08). The results are shown in Fig. 5.

According to the survey, only slightly more than half of the respondents would feel equally sure to indicate endoscopic treatments (n = 30, 65.2%) or surgical procedures (n = 29, 63.04%) both for achalasia and EGJOO.

## Discussion

The results of this study, aimed to provide a real-life snapshot of achalasia spectrum disorders management in the national landscape, revealed a significant heterogeneity across different Italian centers. This disparity could be attributed to the high costs associated with these technologies, which include specialized instrumental equipment, training of dedicated personnel, and structural expenses. On the other hand, these procedures often lack a specific regional coding that ensures reimbursement from the National Health System, resulting in financial burdens for the Healthcare companies. This has limited the practical application and dissemination of diagnostic and therapeutic methods for achalasia spectrum disorders across the centers. Consequently, clinical decisions could be influenced by the differences in available resources at each center.

There are several possible therapeutic options for achalasia; the decision on which type of treatment to offer each patient depends on a reasoned balancing of the expected efficacy, durability of the results, and the risk of complications. Overall, Heller myotomy combined with Dor fundoplication is established as the first-choice procedure in most achalasia cases in patients fit for surgery. However, other less invasive endoscopic procedures, such as BTX and PD, are still considered the first therapeutic attempts for elderly and frail patients.

The results of this survey indicate that there are different therapeutic approaches depending on the achalasia subtype. For type I and II achalasia, in addition to H–D, which is the preferred treatment, BTX, PD, and POEM are offered according to the clinical characteristics of patients and the availability of the procedure. In type III achalasia, where the LES impairment is associated with esophageal body spasm, even if H–D is still considered the best first approach, there is an increase in the percentage of POEM indications, driven by a reduction in the access to BTX and PD, which have demonstrated limited efficacy in this subtype of achalasia.

Whether to perform H–D over POEM has been intensively debated in recent years. To date, there are no randomized clinical trials directly comparing POEM vs. H–D for achalasia. Many systematic reviews and meta-analyses of observational studies have been conducted to clarify the effectiveness of both procedures for the treatment of achalasia, with conflicting results [7–10]. For instance, Schlottman et al. performed a systematic review and meta-analysis to compare postoperative results, including data from 53 studies (5834 patients) on H–D and 21 studies (1958 patients) on POEM [11]. The authors concluded that both the procedures provided excellent postoperative results on dysphagia resolution, with POEM demonstrating slightly better results than H–D both at 12 months (93.5% vs. 91.0%, p = 0.01) and 24 months (92.7% vs. 90.0%, p = 0.01). However, POEM was associated with an increased risk of developing reflux symptoms (OR 1.69, 95% CI 1.33–2.14, *P* < 0.0001), abnormal pH monitoring (OR 4.30, 95% CI 2.96–6.27, *P* < 0.0001) and erosive esophagitis (OR 9.31, 95% CI 4.71–18.85, *P* < 0.0001) than H–D [11]. Also, Andolfi et al. published a meta-analysis including 20 observational studies comparing clinical outcomes of different treatment modalities for achalasia. According to their results, patients with type II achalasia had the best outcomes both after POEM and H–D; POEM demonstrated to be superior to H–D for type III achalasia, probably due to the possibility of extending the myotomy very proximally along the esophageal body (OR 3.50, CI 1.39–8.77, p = 0.007); on the other hand, the higher rate of reflux after POEM (47.5% vs. 11.1%) needs to be acknowledged before offering this procedure to patients. However, the heterogeneity of follow-up periods and definition of success after the procedure of the included studies could have biased the results of this meta-analysis [12].

Despite published data highlighting the possible clinical benefits of POEM for the treatment of achalasia patients, it remains a secondary option mainly due to GERD concerns and the difficulty for patients to access this procedure. Therefore, to date, POEM has not been fully implemented in the clinical practice in Italy.

Controversies also exist regarding the best approach for end-stage achalasia. The 2018 guidelines of the International Society for Diseases of the Esophagus (ISDE) recommended H–D as the first therapeutic option for mega-esophagus, leaving esophagectomy as a second option in case of treatment failure, due to its high morbidity and mortality [13]. Orlandini et al. performed a systematic review and meta-analysis to assess the efficacy of myotomy for end-stage achalasia. They included 16 articles for a total of 350 patients, of which 74 underwent laparotomy while 276 had a laparoscopic approach. The success rate of H–D myotomy was 76%, the reintervention rate was 12.8%, and postoperative morbidity and mortality were respectively 8% and 0.8% [14]. On the other hand, the role of esophagectomy for end-stage achalasia was investigated by Aiolfi et al., who performed a systematic review and meta-analysis including 8 studies published between 1989 and 2014 for a total of 1307 patients [15]. The majority of patients underwent previous endoscopic and surgical attempts to resolve dysphagia but ultimately underwent esophagectomy for the persistence of symptoms. All the patients were operated with an open approach; the postoperative morbidity ranged from 19 to 50%, and the mortality rate was 0–5.4%. The pooled anastomotic leak rate was 7%, and 27.5% of patients required postoperative endoscopic dilatations [15]. However, no data on the outcomes of minimally invasive approaches for esophagectomy in achalasia patients are available in the literature. The results of our survey are heterogeneous, with 50% of respondents choosing to perform H–D, while 22.7% consider esophagectomy. With the ongoing availability of minimally invasive and robot-assisted surgical alternative approaches, whether to perform esophagectomy as the first approach to end-stage achalasia becomes questioned [16]. In fact, H–D does not resolve the risk of esophageal neoplastic transformation, which is known to be higher in achalasia patients, and surveillance endoscopy reliability to detect early cancers can be lower than expected, due to the frequent presence of food retention despite prolonged fasting. Furthermore, since the esophageal dilatation is not reversible when established, some degree of symptoms, ranging from difficulties in esophageal cleaning to aspiration pneumonia, can occur despite complete surgical myotomy. Finally, in case a subsequent esophagectomy is required, a previous myotomy with fundoplication could impair or complicate the construction of the gastric tube. Therefore, no definitive conclusions can be drawn, and well-designed studies comparing minimally invasive esophagectomy to H–D for end-stage achalasia are warranted.

One of the most significant differences of the CCv4.0 is a refined definition of EGJOO, developed in order to address the over-diagnoses of EGJOO with no clinical implications. The CCv4.0 criteria for EGJOO diagnosis are an Integrated Relaxation Pressure (IRP) elevation both in the primary and secondary position and ≥ 20% swallows with elevated intrabolus pressure in the supine position with evidence of peristalsis (low grade, conditional recommendation) [1]. In addition, according to CCv4.0, all the manometric diagnoses of EGJOO should be considered inconclusive; to fulfill a conclusive diagnosis of EGJOO, clinically relevant symptoms and supportive tests, such as barium esophagogram and/or FLIP, are required. The introduction of more stringent criteria has increased the specificity of EGJOO diagnosis; however, it has also affected the number of diagnosed patients, as many cases that do not fulfill all the criteria are deemed “inconclusive”, posing additional challenges for their management.

There are no precise indications of what should be assessed at supportive investigations to confirm the diagnosis of EGJOO [1]. Several radiological parameters adapted from achalasia patients have been proposed [17–20]. However, none demonstrated to be strong enough to support a conclusive diagnosis of EGJOO alone. According to the results of our survey, the main radiological findings that clinicians take into account to support the diagnosis of EGJOO were the presence of esophageal retention of barium above the esophagogastric junction, narrowing of the medium passage at the esophagogastric junction, and esophageal dilatation. On the other hand, the limited availability of the FLIP technology, which is present in only 13% of the responding centers, has limited its role in the clinical practice of EGJOO management in Italy.

Furthermore, no clear indications regarding the optimal therapeutic management of these patients do exist [1]. This lack of shared recommendations translates into the wide variability among the centers regarding the therapeutic approaches to EGJOO, which is documented by the results of this survey. Spontaneous resolution is reported in the literature in 52–92% of EGJOO patients presenting with mild symptoms, and only small series with heterogeneous results have been published on the results of different therapeutic options for EGJOO [21]. According to a narrative review provided by Zikos et al., the pooled success rates of EGJOO patients were 62.3% for no treatment, 63.6% for BTX, 69.6% for endoscopic dilatation, 71.8% for PD, 100% for POEM (but only one study with 3 patients), 100% for PPI treatment [22]. However, all the studies have been settled according to the Chicago Classification 3.0 criteria and, therefore, were not diagnosed according to the newest CCv4.0 classification. According to the results of our survey, overall, the main approach to EGJOO consists of a cautious clinical observation, reserving invasive procedures only when justified by relevant disabling symptoms.

This study has several limitations. The questionnaire was sent to surgeons from all the national territories afferent to SICE, one of the most important surgical societies in Italy. However, the responders may not be representative of national practices. Responses from surgeons could have been more present than other involved non-surgical figures such as endoscopists and clinicians performing esophageal functional examinations. One response for each participant center was required, but multiple inputs from a single center or responses from different centers of a single institution could occur. Finally, the number of respondents is limited, but in line with the shortage of centers dedicated to the diagnosis and treatment of these infrequent diseases.

In conclusion, the results of this study provide valuable insight into the practical approaches for achalasia and EGJOO in the national landscape. Achalasia spectrum disorders are borderline disorders whose diagnosis and treatment involve clinicians from different specialties, and a multidisciplinary approach in their management is advisable. Clinical guidelines are warranted to provide recommendations and shared decisions for the management of EGJOO, to aid the clinician in the decision-making process of both conclusive and inconclusive cases.

## Data Availability

The data that support the findings of this study are available from the corresponding author upon reasonable request.
